# Supplementary material for: PLOS Genetics 2017 Reviewer and Editorial Board Thank You
Source: PLoS Genet. 2018 Mar 15;14(3):e1007265. doi: 10.1371/journal.pgen.1007265 (PMC5854229; doi:10.1371/journal.pgen.1007265)
Supplement: S1 Guest Editor List — (PDF) [file pgen.1007265.s002.pdf]

PLOS Genetics would like to thank all those who served as a Guest Associate Editor in 2017:

Cory Abate-Shen
Ian R. Adams
Asifa Akhtar
Frank W. Albert
Mark K. Alkema
Richard M. Amasino
James Amatruda
Marc Amoyel
Erik C. Andersen
Michael G. Anderson
Leif Andersson
Alex Andrianopoulos
Heinz Arnheiter
Euan A. Ashley
Suzi Atwell
Karen B. Avraham
Zoya Avramova
Willy M. Baarends
Erika A. Bach
Michel Bagnat
Christopher James Bakkenist
Esteban Ballestar
Linda A. Barlow
Scott Barolo
Rodolphe Barrangou
Andrea Barta
Nick H. Barton
Michael Baym
Tom Beeckman
Oded Beja
William J. Belden
Hugo J. Bellen
Rameen Beroukhim
Needhi Bhalla
Laura Bianchi
Perry E. Bickel
Louise S. Bicknell
Anja-Katrin Bielinsky
James A. Birchler
Jesse D. Bloom
Alessandra Boletta
Justin O. Borevitz
Vladimir Botchkarev
Teresa Bowman
Axel A. Brakhage
Rachel B. Brem
Rachel Brem
Sharon Browning
Anne Brunet
Maja Bucan
James J. Bull
Joseph D. Buxbaum
Kerstin Bystricky
Ken M. Cadigan
Kim A. Caldwell
Olivia Casanueva
Tamara Caspary
Amy Caudy
Giacomo Cavalli
Brian P. Chadwick
Debabrata Chakravarti
Fred Chang
Kathryn Song Eng Cheah
Ping Chen
Wei Chen
Alice Cheung
Mordechai Choder
David C. Christiani
Stirling Churchman
Amander Clark
Leigh Anne Clark
David Clayton
Francesca Cole
Karen Conneely
Tim F. Cooper
Vaughn S. Cooper
Jukka Corander
Justin Courcelle
Leah E. Cowen
Allen Cowley

Dana C. Crawford
Chad Creighton
Sean Crosson
Paul J. Cullen
Peter D. Currie
Giovanni D'Angelo
Dean Dawson
Peter de Knijff
Olivier Delattre
Arshad Desai
Luciano Di Croce
Marc I. Diamond
Benjamin W. Domingue
Timothy J. Donohue
Dale Dorsett
Bruce W. Draper
Thomas Dresselhaus
Sarah J. Dunstan
Ian Dworkin
Todd L. Edwards
Michael B. Eisen
Stephen C. Ekker
JoAnne Engebrecht
Xavier Estivill
Elena Ezhkova
Daniel Falush
Christian Fankhauser
Judith Favier
Eric R. Fearon
Edward James Feil
Daniele Filiault
Judith Fischer
Reinhard Fischer
David R. FitzPatrick
Jose C. Florez
Uta Francke
David A. Frank
Hunter B. Fraser
Chris Fromme
Sabine Fuhrmann
Jennifer C. Fung
Patrick M. Gaffney
Li Gan
David Gatfield
Stefan Gaubatz
Anja Geitmann
Arthur Georges
Ali G. Gharavi
Nick Gilbert
Wendy V. Gilbert
Santhosh Girirajan
Jonathan Gitlin
N. Louise Glass
Beverley Glover
Mark Gomelsky
Jose Luis Gomez-Skarmeta
Elena O. Gracheva
David C. Grainger
Michael Granato
Simon Gravel
David Greenstein
Gerard Cornelis Grosveld
David Hafler
Christopher A. Haiman
Hakon Hakonarson
Joachim Hallmayer
Robert S. Haltiwanger
Hiroshi Hamada
Christopher Martin Hammell
Prasada Rao Hanumanthu
Anne C. Hart
Bassem Hassan
Michelle Hastings
Cole Haynes
Domingos Henrique
Yann Herault
Alan J. Herr
Claudio Hetz
Heinz Himmelbauer
Christ Todd Hittinger
Robert Holmgren
Ben F. Holt
Jonathan Houseley
Yariv Houvras
Jian Hua
Adam L. Hughes
Timothy Humphrey
Michael Ibba

Alexander Idnurm
Myron S. Ignatius
Toshiro Ito
Ursula H. Jakob
Richard G. Jenner
Jonathan D. G. Jones
Monica J. Justice
Julie L. Kadrmas
Brendan J. Keating
Scott Keeney
Nancy P. Keller
William G. Kelly
Linda J. Kenney
Andrew Kern
Rene F. Ketting
James Kijas
Stuart K. Kim
Kristina Kirschner
Michael S. Kobor
Tetsuya Kojima
Hisato Kondoh
Raphael Kopan
Rudolf Korinthenberg
Helmut Kramer
Klas Kullander
Justin Kumar
Anshul Kundaje
Edmund Kunji
Albert R. La Spada
Dolores Lamb
Dudley W. Lamming
Amanda Larracuente
Michael T. Laub
Siu Sylvia Lee
Soo Chan Lee
Won-Jae Lee
Michael Lenhard
Richard E. Lenski
Guillaume Lettre
Michael Levine
Mark Lewandoski
Jennifer D. Lewis
Bingshan Li
Jin Billy Li
Jun Li
Yun Li
Hongxuan Lin
Xihong Lin
Marston Linehan
Brian A. Link
Yi Liu
Kirk E. Lohmueller
Stuart J. Macdonald
Dixie L. Mager
Paul M. Magwene
Kateryna D. Makova
Anna Malkova
James Mallet
Francis Martin
Luciano Matzkin
Martin M. Matzuk
John McCutcheon
John C. McDermott
Bruce McEwen
James A. McNew
Brian McStay
Richard Meehan
Cathryn S. Mellersh
Eric M. Mendenhall
Alexey J. Merz
Philipp W. Messer
Blake C. Meyers
Matthew L. Meyerson
Anthony Millar
Jonathan Millar
Aaron P. Mitchell
Elena Monte
Anne M. Moon
Adrian Walton Moore
Ann C. Morris
Naomi S. Morrissette
Ivan P. Moskowitz
Juerg Mueller
Sabine Mueller
John Isaac Murray
Adele Murrell
Kim E. Nichols
Marcelo A. Nobrega

Markus M. Noethen
Roel Nusse
Sergey V. Nuzhdin
Stacey K. Ogden
Guillermo Oliver
David Oppenheimer
Melanie Ott
David C. Page
Ravishankar Palanivelu
Leo J. Pallanck
Martin Parniske
Wojciech P. Pawlowski
Zhen-Ming Pei
Catherine L. Peichel
Jose R. Penades
Sarah Pendergrass
Gislene Pereira
Matthias Peter
Ulrike Peters
Brandon L. Pierce
Corné M.J. Pieterse
Tatjana Piotrowski
Roger Pique-Regi
Joshua B. Plotkin
R. Scott Poethig
Martin Polz
Marcel Quint
Akhila Rajan
Sohini Ramachandran
Carolyn Rasmussen
Andrew Fraser Read
Donald Ready
Jason Reed
Paulo Ribeiro
David P. Rice
Lynn M. Riddiford
Michael Ristow
Steven Roberts
Avital A. Rodal
Antonis Rokas
Joel H. Rothman
Claire Rougeulle
June L. Round
Kirsten Sadler Edepli
Iris Salecker
Silvio Salvi
Saumendra N. Sarkar
Manfred Schartl
Andreas Schedl
John C. Schimenti
Jeremy Schmutz
Richard A. Schneider
Julian I. Schroeder
Danja Schünemann
Trudi Schupbach
Erwin Schurr
François Schweisguth
Amita Sehgal
Michael F. Seldin
Vijay Setaluri
Cynthia M. Sharma
Andrew D. Sharrocks
Val C. Sheffield
Jianxin Shi
David Shore
Lyle A. Simmons
Kathy Sivils
Rolf I. Skotheim
Ian Small
Sarit Smolikove
Rosangela Sozzani
Duncan Sproul
Didier Y. R. Stainier
Tatjana Stankovic
Zornitza Stark
Catherine M. Stein
Alessandro Stella
John Stinchcombe
Petar Stojanov
Francesca Storici
Lucia Strader
Daniel O. Stram
Susan Suarez
Peter E. Sudbery
Xin Sun
Shamil R. Sunyaev
Kankshita Swaminathan
Patrick Tan

Derk ten Berge
Kathrin Thedieck
Irma Thesleff
Timothy A. Thornton
Carl S. Thummel
Paul A. Trainor
Heather L. True
Gosia Trynka
Mick F. Tuite
Tamir Tuller
James M. A. Turner
Paul E. Turner
Juan M. Vaquerizas
Patrik Verstreken
Pablo E. Visconti
Jörg Vogel
Thomas F. Vogt
Marian Walhout
Graham C. Walker
Lori L. Wallrath
Da-Zhi Wang
Meng Wang
P. Jeremy Wang
Mark E. Warchol
Matthew T. Webster
Detlef Weigel
Robert A. Weinberg
Brant M. Weinstein
Mitchell J. Weiss
Ellen Wijsman
Karl Willert
Michael Wilson
Melissa A. Wilson Sayres
Mariana Federica Wolfner
Yannick Wurm
Ting Xie
Jin-Rong Xu
Wei Yan
Jing Yang
Jun J. Yang
Xin Yuan
Noah Zaitlen
Lin Zhang
Hongyu Zhao
Stephan Zuchner
