# Supplementary material for: PLOS Genetics 2017 Reviewer and Editorial Board Thank You
Source: PLoS Genet. 2018 Mar 15;14(3):e1007265. doi: 10.1371/journal.pgen.1007265 (PMC5854229; doi:10.1371/journal.pgen.1007265)
Supplement: S1 Reviewer List — (PDF) [file pgen.1007265.s003.pdf]

PLOS Genetics would like to thank all those who reviewed on the behalf of the journal in 2017:

Pierre Abad
Alejandro Aballay
Omar Abdel Wahab
Sören Abel
Herman Aberle
Alexej Abyzov
Susan Ackerman
Martin Ackermann
Cheryl Ackert-Bicknell
Ian Adams
Mark Adams
Teresa Adell
Sarah Ades
Sankar Adhya
Mateus Adolfi
Stein Aerts
Markus Affolter
Deepa Agashe
Takashi Agui
Pablo Aguilar
Andrés Aguilera
Nadav Ahituv
Nihal Ahmad
Shawn Ahmed
Michael Ailion
Tauqeer Alam
Eric Alani
Qais Al-Awqati
Urs Albrecht
Joy Alcedo
Rebeca Aldunate
Mattias Alenius
Juan Alfonso
Jauhar Ali
Patrick Allard
Tamara Alliston
Jacob Almagro-Garcia
Scott Alper
Bassem Al-Sady
Paulo Amaral
Jorge Eduardo Amaya Romero
Victor Ambros
Gustav Ammerer
Alfred Anambua
Olga Anczukow-Camarda
Bogi Andersen
Carol Anderson
Matthew Anderson
Tim Anderson
Dan Andersson
Göran Andersson
Leif Andersson
Alex Andrianopoulos
Peggi Angel
Montserrat Anguera
Oscar Aparicio
Luís Aragón
Natalia Araujo
Michelle Arbeitman
Benoit Arcangioli
Manuel Ares
Cristiana Argueso
Robert Arkowitz
Arthur Arnold
Brian Arnold
Swathi Arur
Pere Arús
Hiroshi Asahara
Sassan Asgari
Ruth Ashery-Padan
Neil Ashton
Viktor Žárský
William Astle
Paul Auer
Leonard Augenlicht
Juan Ausio
Frédéric Austerlitz
Karen Avraham
Radha Ayyagari
Abdussalam Azem

Veronique Azuara
Willy Baarends
Erika Bach
Joanna Bacon
Luis Alberto Baena-López
Aditya Bagrodia
Melanie Bahlo
Yong-Sun Bahn
Hua Bai
Sheila Baker
Jeroen Bakkers
Sureshkumar Balasubramanian
Darius Balciunas
David Balding
Jillian Banfield
Danika Bannasch
Fernando Baquero
Stefan Barakat
Daniel Barbash
Marie Barberon
Marco Barchi
Allison Bardin
Luca Bargelloni
Fredy Barneche
Scott Barolo
François-Xavier Barre
Luis Barreiro
Jeffrey Barrett
Antoni Barrientos
Gregory Barsh
Julia Barthold
Thomas Bartnikas
Kerstin Bartscherer
Evelina Basenko
Greg Bashaw
Tobias Baskin
Konrad Basler
Brenda Bass
Hank Bass
Diane Bassham
Alexander Bassuk
Analabha Basu
Tuncay Baubec
Amelie Baud
Lars Baumbusch
Iliana Baums
Diana Bautista
Ivan Baxter
Vafa Bayat
Elizabeth Bayne
Ozgur Bayram
Dominik Beck
Anke Becker
Catherina Becker
Claude Becker
Thomas Becker
Antonio Bedalov
Thomas Begley
Susanta Behura
Greg Beitel
Douglas Bell
Alfonso Bellacosa
Hugo Bellen
Mathias Beller
Deborah Bell-Pedersen
Jennifer Below
Bill Bement
Richard Bennett
Matthias Benoit
Yehuda Ben-Shahar
Andrew Bent
Kerin Bentley
Richard Benton
Matteo Bergami
J. Andrew Berglund
Andreas Bergmann
Pascal Bernard
Sonja Berndt
Thomas Bernhardt
Jimena Berni
Kara Bernstein
Maria Celia Bertolini
Alison Bertuch
Joseph Besharse
Florence Besse
Dany Beste
Penny Beuning

Needhi Bhalla
Suvendra Bhattacharyya
Alessandro Bianchi
Piero Bianco
Jason Bielas
Nicolas Bierne
Laura Bierut
Brad Binder
He Bing
Emanuele Biondi
James Birchler
Johan Bjorkegren
Brian Black
T. Keith Blackwell
Alexandre Blais
Garry Blakely
Randy Blakely
Jill Blankenship
Guy Bloch
A. Joseph Bloom
Jesse Bloom
Kerry Bloom
Michael Blower
Michael Blum
Justin Blumenstiel
Tom Blumenthal
Shelby Blythe
Scott Boden
Gregory Boel
Paolo Boffetta
Laszlo Bogre
Dirk Bohmann
Brigitte Boizet-Bonhoure
Michael Boland
Ewelina Bolcun-Filas
Aureliano Bombarely
Peter Bommert
Nina Bonekamp
Stefan Bonn
Jeremy Bono
Daniel Bopp
Guntram Borck
Nica Borgese
Joerg Bormann
Alex Bortvin
Helena Boshoff
Yohan Bosse
Janette Boughman
Guillaume Bourque
Paul Boutros
Paola Bovolenta
Bruce Bowerman
Josephine Bowles
Primrose Boynton
Adrian Bracken
Ingrid Braenne
Sabine Brantl
Gloria Brar
Ana Bratic
Hiltrud Brauch
Andrew Braun
Sigurd Braun
Erin Bredeweg
Alexander Brehm
Rachel Brem
Erhard Bremer
Darren Brenner
Michael Brenowitz
Gregory Brent
Emery Bresnick
Christopher Brett
Rachel Brewster
Miguel Brieno-Enriquez
Henrik Bringmann
Matthias Brock
Susan Brockerhoff
Gudrun Brockmann
Peter Brodersen
Edward Brodkin
Veronique Brodu
Steven Brody
Heather Broihier
Karl Broman
Roland Brosch
Jan Brosens
Susan Broughton
Carolyn Brown
Christopher Brown

Eric Brown
Grant Brown
J. Mark Brown
Kevin Brown
Pamela Brown
Susan Brown
Sharon Browning
Martina Brueckner
Jennifer Brum
William Brunken
Michael Brunner
Kasia Bryc
Robert Bryson-Richardson
Guojun Bu
Esteban Buchard
Nicholas Buchler
Angus Buckling
Bruce Budowle
C. Robin Buell
Avelino Bueno
Alex Buerkle
Mariano Buffone Buffone
Bernd Bukau
Davide Bulgarelli
James Bull
Hannes Bülow
Stephen Buratowski
Emanuele Buratti
Alexandre Bureau
Harold Burgess
Sean Burgess
Shawn Burgess
Daniel Burke
Kristopher Burkewitz
Margit Burmeister
Kathleen Burns
Benjamin Burrows
Vincent Burrus
Briana Burton
Stephen Busby
William Bush
Pierre Bushel
Roger Butlin
Laura Buttitta
Joel Buxbaum
Bernadette Byrne
Chen-Leng Cai
Francesc Calafell
Erin Calfee
George Calin
Brian Calvi
Gerard Campbell
Judith Campbell
Peter Campbell
Jose Cancelas
Sharon Cantor
Blanche Capel
Gabriel Capellá
Maya Capelson
Aurélien Capitan
Michael Caplan
Valeria Capra
Mattia Capulli
Rut Carballido-Lopez
Valerio Carelli
Örjan Carlborg
Sarah Carmona
Marina Carpinelli
Jason Carroll
Gregory Carter
Jaime Carvajal
Jorge Casal
Olivia Casanueva
Tamara Caspary
Stephane Castel
Valerie Castellani
Maria Castro
Amy Caudy
Mathilde Causse
Isabella Ceccherini
Petr Cejka
Craig Ceol
Enrique Cerdá-Olmedo
Maria Fernanda Ceriani
Volkan Cevik
Richard Chahwan
Ankur Chakravarthy
Samya Chakravorty

Kin Chan
Sriram Chandrasekaran
Guillaume Chanfreau
Chenbei Chang
Howard Chang
Michael Chang
Sandy Chang
Yin Chang
Jenny Chang-Claude
Brad Chapman
Michel Chapuisat
Brian Charlesworth
Deborah Charlesworth
Daniel Chase
Daniel Chasman
Dhruba Chattoraj
Jayanta Chaudhuri
Kathryn Cheah
Frédéric Chédin
Iain Cheeseman
Changbin Chen
Chia-Yen Chen
Ching-Kang Jason Chen
Eleanor Chen
Guang-Chao Chen
John Chen
Lin Chen
Liuh-Yow Chen
Wei-Min Chen
Xiaoshu Chen
Z. Jeffrey Chen
Alfred Cheng
Yury Chernoff
Charleston Chiang
Timothy Chico
Cheng-Ting Chien
Rayan Chikhi
Patrick Chinnery
Ian Chin-Sang
Charles Chiu
Joanna Chiu
Queelim Ch'ng
Ken Cho
Keith Choe
Yit-Heng Chooi
Joanne Chory
Clement Chow
King Chow
Wolverton Chris
Brock Christensen
Lionel Christiaen
Peter Christie
Zofia Chrzanowska-Lightowlers
Zhaohui Chu
Cheng-Ming Chuong
Edward Chuong
Victor Cid
Olivier Cinquin
Peter Claes
Steven Clapcote
Andrew Clark
Karl Clark
Leigh Clark
Catherine Clarke
Chris Clarkson
Anne-Kathrin Classen
Stephan Clemens
Wilson Clements
Hans Clevers
Jean-François Cloutier
Jennifer Cobb
Paul Cobine
Peter Cockerill
Paul Cohen
Antonio Colavita
Douglas Cole
Francesca Cole
Jeff Cole
Jerome Collignon
Marco Colonna
Luca Comai
Christophe Combet
Gavin Conant
Ciarán Condon
Frank Conlon
William Cookson
Julia Cooper

Anita Corbett
Victor Corces
Brendan Cormack
Robert Cornell
François Cornet
Montserrat Corominas
Francis Corson
Victoria Cortessis
Frank Costantini
Gilbert Cote
Jacques Côté
George Coupland
Juan Pablo Couso
Leah Cowen
Michael Cox
Timothy Cox
Robert Cramer
Nigel Crawford
Bernard Crespi
James Crichton
Richard Cripps
John Crispino
Sean Crosson
Nicholas Croucher
William Crowley
Damian Crowther
Carlos Cruchaga
Györgyi Csankovszki
Pilar Cubas
Hongchang Cui
Rutao Cui
Paul Cullen
Molly Cummings
M. Joan Curcio
Sean Curran
Patrick Curtis
Asher Cutter
Piotr Czubkowski
Aparecido da Cruz
Alain Dabdoub
Malgorzata Daczewska
Fabrizio d'Adda di Fagagna
Patricia Dahia
Christian Dahmann
Fabrice Danjou
Jeremy Dasen
Arko Dasgupta
Josephine Daub
Vincent Daubin
Alan Davidson
Brad Davidson
Tamara Davis
Subhajyoti De
Valerie de Crécy-Lagard
Stefan de Folter
Cristina de Guzman Strong
Steven de Jong
Jesus de la Cruz
Bernard de Massy
Diego de Mendoza
Joaquin de Navascues
Katleen De Preter
Lieven De Veylder
Joris de Wit
James DeCaprio
Ronny Decorte
Thomas Defalco
Michael DeGiorgio
Filippo Del Bene
Jean Delabar
Olivier Delaneau
Joe Delaney
Miguel De-Lucas
Francesco Demayo
Florence Demenais
Ayse Demirkan
Tanneke den Blaauwen
Christophe d'Enfert
Angela DePace
Brent Derry
Arshad Desai
Eileen Devaney
Olivier Devuyst
Didier Devys
Alessia Di Nardo
Xianmin Diao
Mederic Diard
Charna Dibner

Dion Kai Dickman
Martin Dickman
Yoan Diekmann
Stephen Difazio
Savithramma Dinesh-Kumar
Jonathan Dinman
Sharon Diskin
Christine Disteche
Michael Dixon
Ron Do
Bradley Doble
Angelika Doetzlhofer
Dan Doherty
David Dolezel
Sara Domingues
Maria Dominguez
Xinnian Dong
John D'Orazio
Alessandro Doria
Dale Dorsett
Richard Dorsky
Daolong Dou
Michael Downey
Hermanus Henricus Maria Draisma
Bruce Draper
Cathy Drennan
Wolfgang Driever
Marc Drolet
Tom Druet
Daniela Drummond-Barbosa
Jubao Duan
Qing Duan
Dharani Dubey
Wolfgang Dubiel
Jean-François Dubremetz
Daniel Ducat
Frank Dudbridge
Martin Duennwald
Janet Duerr
Henry Duff
Gaurav Dugar
Stefanie Dukowic-Schulze
Kent Duncan
Donncha Dunican
Jay Dunlap
Delphine Duprez
Adam Dupuy
Raúl Duran
Laurent Duret
Julien Dutheil
Alex Duval
Jonathan Dworkin
Johann Eberhart
Ingo Ebersberger
Richard Ebright
Isaac Edery
Bruce Edgar
Owain Edwards
Stacey Edwards
Idan Efroni
Dan Ehninger
Ian Ehrenreich
Steve Eichten
Eli Eisenberg
Aziz El Hage
Marie Elliot
Chris Elliott
David Elliott
Justine Ellis
Steven Ellis
Patrick Emery
Thierry Emonet
David Enard
Toshiya Endo
JoAnne Engebrecht
James Engel
Nora Engel
Bevin Engelward
Ian Eperon
Sevinc Ercan
Joachim Ernst
Robert Ernst
Ananias Escalante
Valentina Escott-Price
Ragnhild Eskeland
Olivier Espeli
Lucie Etienne

Thomas Eulgem
Ben Evans
Todd Evans
Collin Ewald
Adam Eyre-Walker
Benjamin Ezraty
Maria Falkenberg
Daniel Falush
Steven Farber
Margarida Fardilha
Laurent Fasano
Sébastien Faucher
Michael Federle
Eleanor Feingold
Laura Fejerman
Wenyi Feng
Tim Fenton
Denise Ferkey
Donna Fernandez
Pedro Fernandez-Funez
Alisdair Fernie
Laura Ferraiuolo
Cristina Ferrandiz
Dominique Ferrandon
Miguel Ferreira
Michael Fessing
Paul Fey
Gabriella Ficz
Aretha Fiebig
Janna Fierst
Duarte Figueiredo
Iwona Fijalkowska
Daniele Filiault
Guillaume Filion
John Fingert
Ruth Finkelstein
Carla Finkielstein
Silvia Finnemann
Amanda Fisher
Shannon Fisher
David Fitzpatrick
Frederic Flamant
Nuria Flames
Paul Flicek
Eric Folker
Michaela Fontenay
Anja Forche
James Ford
Fabio Fornara
Ian Forster
Alexandre Fournier-Level
Catherine Fox
Don Fox
Michael Francis
C. Andrew Frank
Hunter Fraser
James Fraser
Catherine Freudenreich
Steven Friedenberg
Angela Friederici
Timothy Friesen
Maxim Frolov
Judith Frydman
Masaya Fujita
Tatsuo Fukagawa
Melissa Fullwood
Matteo Fumagalli
Jennifer Fung
Eileen Furlong
Toni Gabaldon
Davide Gabellini
Olivier Gadal
Fred Gage
Kimberly Gallagher
Peter Gallant
Maria Gallegos
Maria-Trinidad Gallegos
Romain Gallet
Ohad Gal-Mor
Fei Gao
Fen-Biao Gao
David Garfinkel
Lana Garmire
Peter Garred
Jennifer Garrison
Anton Gartner
Audrey Gasch
Charles Gasser

Susan Gasser
Reto Gassmann
David Gatfield
Eric Gaucher
Brandon Gaut
Mackenzie Gavery
Simon Gayther
Brian Gebelein
Vincent Geli
Diane Genereux
Pierre Genevaux
Rita Gerardy-Schahn
Arjumand Ghazi
Anindya Ghosh-Roy
Amato Giaccia
Claudia Giambartolomei
Marios Giannakis
Richard Gibbons
Keylie Gibson
Danna Gifford
Christopher Gignoux
Matthew Gilliham
Reid Gilmore
Anne-Paule Gimenez-Roqueplo
Luca Giorgetti
James Giovannoni
Chloe Girard
Aaron Gitler
Christos Gkogkas
Pierre Gladieux
Philippe Glaser
N. Louise Glass
Jane Glazebrook
Christopher C. Glembotski
Beverley Glover
Mark Glover
Sarah Goetz
Amy Goldberg
Erin Goley
Pierre Goloubinoff
Mark Gomelsky
Jose Luis Gomez-Skarmeta
Qizhi Gong
Zhizhong Gong
Benjamin Good
Jeffrey Good
John Goodier
Justin Goodrich
Stephen Gordon
Harald Goring
Florien Gorter
Gohta Goshima
Monica Gotta
Fred Gould
Mark Goulian
Pravitt Gourh
Campbell Gourlay
Mina Gouti
Francine Govers
Humaira Gowher
Todd Graham
Ian Grainge
David Grainger
Muriel Grammont
Michael Granato
Barth Grant
Struan Grant
Peter Graumann
Simon Gravel
Stephen Gray
Casey Greene
Andy Greenfield
Ralph Greenspan
Eric Greer
T. Ryan Gregory
David Gresham
Joachim Griesenbeck
Yevgenya Grinblat
Anthony Griswold
Amel Gritli-Linde
Kathryn Grive
Carol Gross
Christina Gross
Jörg Grosshans
Christina Grozinger
Rita Groβ-Hardt
Stephan Gruber

Weifeng Gu
Min-Xin Guan
Marc-Jan Gubbels
Daniel Gudbjartsson
Renzo Guerrini
Roderic Guigo
Yann Guiguen
Guillaume Guilbaud
Hervé Guillou
Barry Gumbiner
Bhagwati Gupta
Sunetra Gupta
Stephanie Gupton
John Gurdon
Victor Guryev
Claes Gustafsson
Crisanto Gutierrez
Lionel Guy
Hubertus Haas
James Haber
Michael Habig
Udo Haecker
Wilfried Haerty
Pejmun Haghighi
Matthew Hahn
Neena Haider
David Haig
Alex Hajnal
Sven Halbedel
Randal Halfmann
Brandon Hall
Ruth Hall
Michael Hallett
Benedikt Hallgrimsson
Marnie Halpern
Scott Halstead
Christopher Hammell
Molly Hammell
John Hammond
Iqbal Hamza
Buhm Han
Fangpu Han
Lynn Hancock
Mary Ann Handel
Joseph Hanly
Wendy Hanna-Rose
Abdelali Hannoufa
Anders Hansen
David Hansen
Susan Harbison
Alison Hardcastle
Paul Hardin
J. Marie Hardwick
John Hardy
Arbel Harpak
Kelley Harris
Douglas A. Harrison
Elizabeth Harry
John Hartman
Grant Hartzog
Natasha Harvey
Mike Hasegawa
Bret Hassel
Yutaka Hata
Shin Hatakeyama
Felix Hauser
Susanne Häussler
Thomas Hawn
Jesse Hay
Cole Haynes
Ping He
Xi He
Xin He
Xin-Jian He
Yuke He
Aoife Heaslip
Joan Heath
Madhuri R. Hedge
Iris Heid
Lutz Hein
Yrjo Helariutta
Stefan Heller
Dominique Helmlinger
Martin Hemberg
Ian Henderson
Brenna Henn
Clarissa Henry
Yann Herault

Julie Herbstman
Jennifer Herman
Amaury Herpin
Eloisa Herrera
Ronna Hertzano
Doris Herzlinger
Rex Hess
Jody Hey
Ahlke Heydemann
Wolf-Dietrich Heyer
Mark Hickman
Meleah Hickman
Alicia Hidalgo
Robin Hiesinger
Ville Hietakangas
N. Patrick Higgins
Doug Higgs
Geoffrey Hill
Angie Hilliker
Gary Hime
Philip Hinchliffe
Justin Hines
Noboru Hiroi
Candice Hirsch
Jay Hirsh
Nobuyuki Hizawa
Lawrence Hobbie
Andreas Hochwagen
Charles Hoffman
Kay Hofman
Ben Hogan
John Hogenesch
Saskia Hogenhout
Christer Hogstrand
Peter Hohenstein
Seamus Holden
Nancy Hollingsworth
Erik Holmqvist
Ben Holt
Marina Holz
Christian Hong
Christer Höög
Tim Hore
Tomoaki Horie
Sally Horne-Badovinac
Rita Horvath
Jianghui Hou
Gunnar Houge
Jonathan Houseley
Stephen Howell
Niall Howlett
Hsu-Liang Hsieh
Ao-Lin Hsu
Hwei-Jan Hsu
Patrick Hu
Yi-Juan Hu
Yuxin Hu
Guanghua Huang
Hailiang Huang
Kaiyao Huang
Kerwyn Huang
Sanwen Huang
Xuehui Huang
Armin Huber
Martin Hudson
Emilia Huerta-Sanchez
Andrew Huh
Jin Hoe Huh
Christina Hull
David Hume
Steven Hunt
Enamul Huq
Adam Hurlstone
Patrick Hussey
Nancy Hynes
Pirro Hysi
Kenji Ichiyanagi
Oleg Igoshin
Masahito Ikawa
Akihiro Ikeda
Sakae Ikeda
Yuzuru Imai
Takato Imaizumi
Axel Imhof
James Imlay
Hanne Ingmer
Nicholas Ingolia
Ken Inoki

Kunio Inoue
Satoshi Inoue
Iuliana Ionita-Laza
Nicola Iovino
Y. Tony Ip
Kenneth Irvine
Yuval Itan
Cristina Ivan
Hiroshi Iwasaki
Shigeki Iwase
Shintaro Iwashita
Sangeetha Iyer
David Jackson
Ian Jackson
Suzanne Jacobs
Andrew Jaffe
Mattias Jakobsson
Hsieh James
Timothy James
Guilhem Janbon
Lars Jansen
Marnix Jansen
Isabelle Jariel
Dan Jarosz
Heinrich Jasper
Ralf Jauch
Hyunsoo Je
Daniel Jeffares
Albert Jeltsch
Choongwon Jeong
Loydie Jerome-Majewska
Suresh Jesuthasan
Dongyu Jia
Jin Jiang
Francis Jiggins
Yishi Jin
Christian C. Jobin
Mark Jobling
Elizabeth Jockusch
Hanna Johannesson
Kristen Johansen
Alexander Johnson
Kenneth Johnson
Welkin Johnson
Nils Johnsson
Robert Johnston
William Joiner
Kristina Jonas
Jeff Jones
Peter Jones
Matthieu Joosten
Craig Jordan
Philip Jordan
Lynn Jorde
Helle Jorgensen
Carolina Jorgez
Emily Josephs
Pradeep Joshi
Luke Jostins
Laurent Journot
Gabor Juhasz
Magdalena Julkowska
Suckjoon Jun
Peter Juo
Steffen Just
Vesa Kaartinen
Mehdi Kabbage
Sebastian Kadener
David Kadosh
Matt Kaeberlein
Henrik Kaessmann
Mark Kahn
Frank Kaiser
Ursula Kaiser
Krisztina Kaldi
Isgouhi Kaloshian
Vera Kalscheuer
Ritsu Kamiya
Malek Kamoun
Martin Kampmann
Ayten Kandilci
Patricia Kane
Hong-Gu Kang
Hyun Min Kang
Min-Ji Kang
Madhuri Kango-Singh
Artur Kania
Patricia Kannouche

Marc Kantorow
Aimee Kao
Craig Kaplan
Marika Kapsimali
Aurélie Kapusta
Mariusz Karbowski
Katrin Karbstein
Elinor Karlsson
Judith Kassis
Fumiaki Katagiri
Vaishali Katju
Jim Kaufman
Matthew Kayser
Stephen Kearsey
Liam Keegan
Alex Keene
Scott Keeney
Peter Keightley
Kenneth Keiler
Gabriella Kelemen
Nancy Keller
Michael Kelly
Thomas Kelly
William Kelly
Timothy Kennedy
Andrew Kern
Rachel Kerwin
Hemant Khanna
Arkady Khodursky
Chiea Khor
Sohail Khoshnevis
Ekta Khurana
Thomas Kidd
Amy Kiger
Masahide Kikkawa
Helena Kilpinen
Byunghyuk Kim
Dennis Kim
Un-Kyung Kim
Yuseob Kim
Koutarou Kimura
Elizabeth King
Mary Lou King
Usha Kini
Mark Kirkpatrick
Antonis Kirmizis
Joseph L. Kirschvink
Sabine Klaassen
Christian Klambt
Eric A. Klein
Hannah Klein
Jurgen Kleine-Vehn
Joel Kleinman
April Kleppe
Daniel Kliebenstein
Andrew Klocko
Robert Klose
Michael Knop
Elisabeth Knust
Junya Kobayashi
Taeko Kobayashi
Daniel Koboldt
Michael Kobor
Lutz Kockel
Theresa Koehler
Vanessa Koelling
Daniel Koenig
Michel Koenig
Ronald Koenig
Hyongjong Koh
Kyunghee Koh
Masaaki Komatsu
Zacharias Kontarakis
Scott Kopetz
Jan Korbel
Philipp Korber
Benoit Kornmann
Nicole Koropatkin
Arthur Korte
Erika Kothe
Peter Kraft
Jordan Kreidberg
Verena Kriechbaumer
Keerthi Krishnan
Arnold Kristjuhan
Bryan Krock
Daniel Kronauer
Joachim Krug

Damian Krysan
Ulrich Kück
Grzegorz Kudla
Christa Kuehn
Anuj Kumar
Justin Kumar
Tsutomu Kume
Markus Kunze
Martin Kupiec
Shigehiro Kuraku
Hidehito Kuroyanagi
Zoltán Kutalik
Kerstin Kutsche
Andrei Kuzminov
June Kwak
Young Kwon
Michael Kyba
Sophie La Salle
Soni Lacefield
Joseph Lachance
Salil Lachke
Michael Ladomery
Thomas Laframboise
Yi Lai
Diana Laird
Roosa Laitinen
Subhash Lakhotia
Teresa Lamb
Sarah Lambert
Louis Lambrechts
Katja Lamia
Dan Landau
Matthias Landgraf
Christian Landry
Carol Lange
David Langenau
Thomas Langer
Gregory Lanzaro
Louis Lapierre
Luis Larrondo
Erica Larschan
Lars Larsen
Lionel Larue
Paul Lasko
Andrew Lassar
Brittany Lasseigne
Jean-Paul Latge
Nelson Lau
On Sun Lau
Michael Laub
Adam Lauring
Jörn Lausen
Elizabeth Lawlor
Michael Lawrence
Daniel Lawson
Nathan Lawson
Jozef Lazar
Yoann Le Breton
Brittany Lee
Dongwon Lee
Grace Lee
Hane Lee
Kristin Lee
Kyu-Sun Lee
Miler Lee
Sang Eun Lee
Seung-Jae Lee
Siu Sylvia Lee
Yin-Won Lee
Peter Leegwater
Louis Lefebvre
Peter Lefebvre
Tony Lefebvre
Véronique Lefebvre
Nele Lefeldt
Ellen Leffler
Michael Lehmann
Ben Lehner
Monkol Lek
Christophe Lemaire
Bernardo Lemos
Alan Leonard
Pierre Leopold
Holger Lerche
Noelle L'Etoile
Guillaume Lettre
Henry Levin
Petra Levin

Mia Levine
Zachary Lewis
Chao Li
Chuan Li
Hongju Li
James Li
Jia Li
Jian-Dong Li
Jigang Li
Jing Li
Jingjing Li
Jun Li
Melody Li
Peng Li
Qi-Jing Li
Shisheng Li
Tiansen Li
Wenbo Li
Xia Li
Xin Li
Yang Li
Yun Li
Yvonne Li
Dezhi Liao
Yi Liao
David Liberles
Susan Liebman
Marjorie Liénard
Robert Lightowlers
Petros Ligoxygakis
Sarah Liljegren
Christopher D. Lima
Jing-Jer Lin
Weichun Lin
Xinhua Lin
Adrian Linacre
Sara Lindström
Jairam Lingappa
Jonathan Lipton
Michael Lisby
James Lister
Tom Little
Andrew Liu
Bo Liu
Chia-Yang Liu
Chunyu Liu
Cuimin Liu
Dajiang Liu
Feng Liu
Hongtao Liu
Hong-Xiang Liu
Mingxi Liu
Mofang Liu
Nianjun Liu
Pingsheng Liu
Sha Liu
Xuezhong Liu
Yaoguang Liu
Yi Liu
Yusen Liu
Bertrand Llorente
Matxalen Llosa
Thomas Lloyd
Kirill Lobachev
Anders Løbner-Olesen
Adam Locke
Michael Lodato
Irfan Lodhi
Ingrid Lohmann
Kirk Lohmueller
Alejandro Lomniczi
Anthony Long
Fanxin Long
Jirong Long
Michelle Longworth
Daniel López
Luis Lopez-Molina
Esben Lorentzen
Oscar Lorenzo
Christian Lorson
Ana Losada
Stephen Loughran
Susan Lovett
Jeffrey Lozier
Jian Lu
Jining Lu
Qiongshi Lu
Weining Lu

Xiaowei Lu
Sheng Luan
Francesca Luca
Neal Lue
Reinhard Lührmann
Brian Luke
Julius Lukes
Andrea Lunardi
Anders Lund
Pete Lund
Peter Lund
Erik Lundquist
Lijun Luo
Wenqin Luo
Shelley Lusetti
Aldons Lusis
Arthur Lustig
Joe Lutkenhaus
Zoi Lygerou
Jeremy Lynch
Vincent Lynch
Dengke Ma
Fen Ma
Hong Ma
Shuyi Ma
Yong-Chao Ma
David MacAlpine
Marcy MacDonald
Deborah Mackay
R. Craig MacLean
Amy MacQueen
Rohan Maddamsetti
Frank Madeo
Morris Maduro
Robert Maeda
Hiromi Maekawa
Dixie Mager
Keith Maggert
Vittorio Maglione
Enrico Magnani
Paul Magwene
Eamonn Maher
Moe Mahjoub
Rafiee Mahmoud-Reza
Ari Pekka Mahonen
Shaun Mahony
Eleanor Maine
William Mair
Pascal Maire
Svetlana Makovets
Jarema Malicki
Jennifer Malin
Anna Malkova
Moises Mallo
Kalle Malmberg
Jacob Malone
John Malone
J. Robert Manak
Pablo Manavella
Eugenio Mancera
Lolitika Mandal
Marco Mangone
Jaan Männik
Suzanne Mansour
Miguel Manzanares
Costas Maranas
Marina Marcet-Houben
Douglas Marchuk
Edoardo Marcora
František Marec
William Margolin
Riccardo Marioni
Manuel Mark
David Markovitz
Luciano Marraffini
Rolf Marschalek
Alexander Marson
Adam Martin
Alicia Martin
Simon Martin
Maria Dolores Martin-Bermudo
Simone Martinelli
José Martínez
German Martinez Arias
Antonio Martínez-Laborda
Enrique Martinez-Perez
Alexandra Martins

Pekka Marttinen
Joanna Masel
Sergei Maslov
Annaliese Mason
Andrew Massey
Ruth Massey
Grant Mastick
Iain Mathieson
Michael Matise
Andreas Matouschek
Tatsuya Matsubara
Minami Matsui
Daniel Matute
Luciano Matzkin
Cedric Maurange
Andreas Mayer
Helen May-Simera
Mona Mazaheri
Didier Mazel
Luke Mccaffrey
David McCandlish
Sarah McClelland
Tim McClintock
Kevin McConway
Mark McCormick
Bruce McDonald
J. Tyson Mcdonald
Michael McDonald
Michael McEachern
Ken McElreavey
Edwina McGlinn
Kelly McGowan
Alistair McGregor
Kim McKim
Jake McKinlay
Richard McLaughlin
Andrew McMahon
James McManaman
Joel McManus
Francis McNally
Helen McNeill
Mary Sara McPeek
Mitch McVey
Kathryn Medler
Paul Medvedev
Richard Meehan
Heather Mefford
Wilfried Meijer
Colin Meiklejohn
David Meinke
Ronald Melki
Cathryn Mellersh
Ralf R. Mendel
Cathy Mendelsohn
Eric Mendenhall
Jerome Menet
Juanita Merchant
Matthias Merkenschlager
Houra Merrikh
Brian Metzger
Axel Meyer
Christian Meyer
Stefan Meyer
Grégoire Michaux
Daniel Michele
James Mickelson
Irene Miguel-Aliaga
Alexander Mikheyev
Marco Milan
Lili Milani
Sarah Millar
Sean Millard
Adam Miller
Craig Miller
Dana Miller
Gary Miller
Ken Mills
Joshua Millstein
Eric Milot
Olga Minkina
Siavash Mirarab
Yuichiro Mishima
Rakesh Mishra
Markus Missler
Charlotte Mistretta
David Mitchell
Hannah Mitchison
Maria Mittag

Pedro Miura
Shuhei Miyashita
Marek Mlodzik
Danesh Moazed
Julius Mojica
Magnus Monné
Dimitri Monos
Cristina Montagna
Jacques Montagne
Stephen Montgomery
Francesco Montinaro
Sally Moody
Jason Moore
Carlos Moraes
Charles Moran
Kevin Morano
Lluis Morey
David Morgan
Peter Morrell
Geoffery Morris
Herbert Morse III
David Morton
Lindsay Morton
Alan Moses
Joseph Mougous
Michelle Mousel
Loukas Moutsianas
Catherine Moyes
Leonie Moyle
Iva Mozgova
Wellington Muchero
Shahid Mukhtar
H. Arno Muller
William Muller
Daniel Mulvihill
Stefan Mundlos
Edwin Munro
Eduard Murani
Yoshiyuki Murata
Kenan Murphy
Johanne Murray
Sean Murray
Adele Murrell
Antonio Musio
Günther Muth
Peter Myler
Kyungjae Myung
Felix Naef
Anja Nagel
Emi Nagoshi
László G. Nagy
Hidewaki Nakagawa
Mike Nalls
Satoshi Namekawa
Maithreyi Narasimha
Dick Nässel
David Natale
Pradeep Natarajan
Francisco Naya
Matthew Neale
Richard Neher
Christoffer Nellaker
David Nelson
Paul Newcombe
Dianne Newman
Joanne Ngeow
Ying Ni
Hannah Nicholas
Michael Nickerson
Conrad Nieduszynski
Timothy Niewold
Hironori Niki
Jakob Nilsson
Ran Nir-Paz
Eisuke Nishida
Hiroki Nishida
Isao Nishimura
Takashi Nishimura
Lee Niswander
Takeshi Noda
Ellen Nollen
Minou Nowrousian
Evgeny Nudler
Todd Nystul
John O'Brien
Richard O'Brien
Donal O'Carroll
Matthew O'Connell

Mitchell O'Connell
Michael O'Connor
Timothy O'Connor
Allyson O'Donnell
Mark O'Driscoll
Matthew Oetjens
Yuya Ogawa
Shuji Ogino
Damien Ohalloran
Tatsuya Ohhata
Benjamin Ohlstein
Hitoshi Okazawa
Snezhana Oliferenko
Carlos Oliva
Brian Oliver
Bradley Olwin
James Olzmann
Daisuke Ono
Christiane Opitz
Michael O'Rand
Marc Orbach
Richard Ordway
Teresa Orenic
Kim Orth
Nir Osherov
Stephen Osmani
Jennifer Ovenden
Katharine Owen
Tom Owen-Hughes
Annalise Paaby
James Padbury
Pamela Padilla
Luca Pagani
Andrea Page-McCaw
Dasaradhi Palakodeti
Javier Palatnik
James Palis
Kelli Palmer
Bernhard Palsson
Quintin Pan
Wei Pan
Ying-Xian Pan
Arun Pandiri
Pier Paolo Pandolfi
Michael Pankratz
Vikram Panse
Nickolas Papadopoulos
Peter Parham
David Parichy
Nuria Paricio
Lani Park
Heidi Parker
J. Alex Parker
J. Brandon Parker
Miles Parkes
Stephen Parnell
Vladimir Parpura
Mathew Parsek
Leopold Parts
Bogdan Pasaniuc
Juan Pascual-Anaya
Amy Pasquinelli
Maria Rita Passos-Bueno
Tomi Pastinen
Andrew Paterson
James Patton
Soumen Paul
Wojciech Pawlowski
Bernhard Payer
Ales Pecinka
Andrew Peden
Itsik Pe'er
Laurence Pelletier
Miguel Peñalva
Giuseppa Pennetta
Marta Perego
J. Christian Perez
Brian Perkins
Steven Perlman
Norbert Perrimon
Laurent Perrin
John Perry
Michael Perry
Rachel Perry
Melissa Pespeni
Ben Peter
Antoine Peters
Heiko Peters

James Peters
Christian Petersen
Craig Peterson
Thomas Petes
Galina Petukhova
Roberto Pezza
Boris Pfander
Gerd Pfeifer
Hemali Phatnani
Megan Phifer-Rixey
Franck Pichaud
Martin Pichler
Leslie Pick
Alisa Piekny
Brandon Pierce
Hugh Piggins
Catherine Pihoker
Manoj Pillai
Marc Pilon
John Pimanda
Manuel Piñeiro
Lionel Pintard
Stefan Pinter
Roger Pique-Regi
Matti Pirinen
Christian Pirk
Antonio Pisani
Steven J. Pittler
Timothy Plageman
Alexander Platt
Anne Plessis
Christian Pohl
Marco Pontoglio
John Pool
Anthony Poole
Richard Poole
David Popham
John Postlethwait
Alex Postma
Christopher Potter
Craig Powell
Madapura Pradeepa
Mónica Pradillo
Vincent Prevot
Alkes Price
David Price
Richard Proia
Daniel Promislow
Molly Przeworski
William Pu
Sergi Puig
Michael Purugganan
George Pyrowolakis
Lihong Qi
Qibin Qi
Xinshaui Qi
Feng Qian
Shu-Bing Qian
Weiqiang Qian
Hong Qiao
Genji Qin
Huaizhen Qin
Jianwen Que
Julia Questa
Francisco Quintana
Fernando Racimo
Sunish Radhakrishnan
Jimmy Rae
Paul Rainey
Joe Rainger
Fabienne Rajas
Yoav Ram
Kumaran Ramamurthi
Alfredo Ramirez
Alessandra Rampazzo
David Rand
Lennart Randau
Thomas Rando
Morten Rasmussen
Aakrosh Ratan
Kristipati Ravi Ram
Aditi Ravindranath
Alan Rawls
Timothy Read
Donald Ready
Ilaria Rebay
Oded Rechavi
Peter Redder

Rosemary Redfield
Floyd Reed
Michael Rehli
Andreas Reichert
Adam Reid
Jochen Reif
Muredach Reilly
Bjorn Reinius
Valerie Reinke
Tânia Reis
Stephanie Reissman
Caroline Relton
Nicolas Renier
Boris Reva
Federico Rey
Rodrigo Reyes Lamothe
Todd Reynolds
Turk Rhen
Amer Riazuddin
Eric Richards
Joan Richtsmeier
Arthuer Riggs
Niels Ringstad
Jürgen Ripperger
Meritxell Riquelme
Michael Ristow
Marylyn Ritchie
Manuel Rivas
Jaime Rivera-Perez
William Rizzo
Benjamin Roa
Chloe Robins
Gene Robinson
Nigel Robinson
Marc Robinson-Rechavi
Maria Robles
Xavier Roca
Sonia Rocha
Matthew Rockman
Eli Rodgers-Melnick
Ramiro Rodriguez
Adrienne Roeder
Kathryn Roeder
Claire Rogel-Gaillard
Andrew Roger
Rebekah Rogers
Gary Rohrer
Ignasi Roig
Jean-Yves Roignant
Antonis Rokas
Micha Ron
Christopher Rongo
Katja Röper
Joanna Rorbach
Mark Rose
David Rosenkranz
Monica Roth
Joel Rothman
Sophie Rousseaux
Simon Roux
François Rouyer
Sushmita Roy
Julien Royet
Ignacio Rubio Somoza
Douglas Ruden
Adam Rudner
Elena Rugarli
Agustín Ruiz
Natividad Ruiz
Aurora Ruiz-Herrera
Edward Ruiz-Narvaez
Laura Rusche
Chris Rushlow
Christine Rushlow
Paul Russell
Ilya Ruvinsky
Gary Ruvkun
Hays Rye
Hyung Don Ryoo
Giuseppe Saccone
Matthew Sachs
Yumiko Saga
Alvaro Sagasti
Jørn Sagen
Megha Sah
Noriko Saitoh
Carlo Saitta
Daisuke Sakai

Suzana Salcedo
Iris Salecker
Leonardo Salviati
Helen Salz
Rodney Samaco
Christos Samakovlis
Yasemin Sancak
Roger Sandhoff
Steven Sandler
Suzanne Sandmeyer
Björn Sandrock
Tzu-Kang Sang
Dominique Sanglard
Subramanian
Sankaranarayanan
Sriram Sankararaman
Simone Santos
Funda Sar
Zanders Sarah
Peter Sarkies
Kavitha Sarma
Hiroyuki Sasaki
Makoto Sato
John-Demian Sauer
Pierre Savatier
Ritwick Sawarkar
James Sawitzke
John Sayer
Ian Sayers
Peter Scacheri
Aylwyn Scally
Michael Scanlon
Enrico Scarpella
Christian Schaaf
Joseph Schacherer
Daniel Schaid
Manfred Schartl
Dirk-Jan Scheffers
Herb Schellhorn
Mark Schembri
Elmar Schiebel
Alexander Schier
Thomas Schilling
John Schimenti
Lisa Schimmenti
Jennifer Schisa
Hilde Schjerven
Klaus Schläppi
Christa Schleper
Peter Schloegelhofer
Karl Schmid
Thomas Schm^{n}lling
Robert Schnabel
James Schnable
Igor Schneider
Kay Schneitz
Frank Schnorrer
Susanne Schoch
Joshua Schraiber
Julian Schroeder
Ingo Schubert
Oren Schuldiner
Stefan Schulte-Merker
Richard Schultz
Fredrick Schumacher
Molly Schumer
Erwin Schurr
Yuri Schwartz
François Schweisguth
Ronen Schweitzer
Benjamin Schwessinger
Daniel Scoles
Ian Scott
William Scott
Paul Scotting
Ralph Scully
Charles Scutt
Julie Secombe
Alisa Sedghifar
Gerhard Seisenbacher
Jeff Sekelsky
Michael Seldin
Anna Selmecki
Julien Seneschal
Kirsten Senti
Seongjin Seo
Giovanna Serino
Mihaela Serpe

Susan Service
Bertrand Servin
Chetan Seshadri
Peter Setlow
Agnel Sfeir
Amanda Sferruzzi-Perri
Orie Shafer
Premal Shah
Shai Shaham
A. Hunter Shain
Libo Shan
Changwei Shao
Michael Shapira
Beth Shapiro
Timothy Francis Sharbel
Cynthia Sharma
Upasna Sharma
Thomas Sharpton
Andrew Sharrocks
Brian Shaw
Paul Shaw
David Shechter
David Sherwood
Jianxin Shi
Qinghua Shi
Toshiharu Shikanai
Ben-Zion Shilo
Jiwon Shim
Toshi Shioda
Assia Shisheva
David Shore
Joel Shore
Weinian Shou
Cheryl Shoubridge
Eric Shoubridge
Daniel Shriner
Elaine Sia
L. David Sibley
Gaganpreet Sidhu
Solveig Sieberts
Derek Sieburth
Weiva Sieh
Daria Siekhaus
Sarah Signor
Martin Sikora
Olin Silander
Jerson Silva
Neal Silverman
Robert Silverman
Martin Simard
Antonio Simeone
Martine Simonelig
Amit Singh
Nadia Singh
Matthias Sipiczki
Jim Skeath
Daniel Skelly
Robert Skoff
Karl Skorecki
Jan Skotheim
Robert Sladek
Matthew Slattery
Stephen Small
Andrei Smertenko
Adrian Smith
Dean Smith
Duncan Smith
Frances Smith
Gerald Smith
Rachel Smith-Bolton
Sarit Smolikove
David Smyth
Kimberley Snowden
Elizabeth Snyder
R. Elizabeth Sockett
Nick Sokol
Jordi Solana
Lilianna Solnica-Krezel
Paul Soloway
David Somers
Ralf Sommer
Imre Somssich
Abraham Sonenshein
Bao-Liang Song
Chun-Peng Song
Poul Sorensen
Alexander Soukas
Vitor Sousa

Rita Sousa-Nunes
Anne Spang
Doug Speed
Johannes Spelbrink
Vanessa Sperandio
Benjamin Spike
Stephen Spiro
Mikhail Spivakov
Simon Sprecher
Duncan Sproul
Supriya Srinivasan
Matthew Stachler
Maike Stam
Remco Stam
Pamela Stanley
Jeremy Stark
W. Marshall Stark
Mike Stear
Catherine Stein
Eirikur Steingrimsson
Lars Steinmetz
Alexandre Stewart
James Stewart
Rodney Stewart
William Stewart
John Stinchcombe
Timothy Stinear
Hugo Stocker
Esther Stoeckli
Peter Stoilov
Angelika Stollewerk
Sophia Stone
Mark Stoneking
Henrik Strahl
Nicola Strenzke
Lena Ström
Joanne Stubbe
Jörg Stülke
Jason Stumpff
Hendrik Stunnenberg
Le Su
Vijayalakshmi Subramanian
Henry Sucov
Katsunori Sugimoto
Jae Hoon Sul
Charlotte Sumner
Lei Sun
Meng-Xiang Sun
Ruping Sun
Wei Sun
Xin Sun
Meera Sundaram
Per Sunnerhagen
Shamil Sunyaev
Michael Sussman
Mark Sutton
Petr Svoboda
Christina Swanson
Maurice Swanson
Sonja Swanson
Colin Sweeney
Sean Sweeney
Andrea Sweigart
Judit Szecsi
Nathaniel Szewczyk
Shunichi Takeda
Yusuke Takehana
Tatsuya Takemoto
Norio Takeshita
Frank Takken
Toru Takumi
Bao-Cai Tan
Minoru Tanaka
Motomasa Tanaka
Shigeyuki Tanaka
Guy Tanentzapf
Haixu Tang
Nan Tang
Toshiyasu Taniguchi
Kandice Tanner
Mark Tanouye
Ran Tao
Aaron Tarone
Marco Tartaglia
Murat Tasan
Marc Tatar
Nektarios Tavernarakis
Dan Tawfik

Guy Tear
Maria Teresa Teixeira
Miguel Teixeira
Aurelio Teleman
Aurélien Tellier
Liesbet Temmerman
Maud Tenaillon
Chong Teng
Andrew Teschendorff
Jens Tetens
Jitendra Thakur
Martin Thanbichler
Nishant Thazath
Douglas Theobald
Pascal Therond
Johan Thevelein
Dennis Thiele
David Thomas
Charles Thompson
Stefan Thor
Joseph Thornton
Timothy Thornton
Peter Tiffin
Marcel Tijsterman
Fausto Tinti
Laurent Tiret
Peter Todd
Seiichi Toki
Gabriela Toledo-Ortiz
David Tollervey
Nicholas Tolwinski
Yukihide Tomari
Kazuhito Tomizawa
Andrew Tomlinson
Craig Tomlinson
Eva Top
Ivan Topisirovic
Miguel Torres
Jesus Torres-Vazquez
Federico Torta
Attila Toth
Aminata Toure
Jacquetta Trasler
Michael Travisano
David Tremethick
Timothy Triche
Frédéric Tripet
Emily Troemel
Melissa Troester
Felix Tropf
John Trowsdale
Heather True
Marco Trujillo
Ala Trusina
Miltos Tsiantis
Leonidas Tsiokas
Noriyuki Tsumaki
Susan Tsunoda
Zhijian Tu
Mark Tucker
Cristina Tufarelli
Taru Tukiainen
Shripad Tuljapurkar
Michael Turelli
Peter Turnbaugh
Doug Turnbull
Eric Turner
James Turner
Baris Tursun
Brett Tyler
Yehuda Tzfati
Thomas Ulbright
Igor Ulitsky
Roman Ulm
Helle Ulrich
Robert Unckless
Nobuyuki Uozumi
Dustin Updike
Sylvie Urbe
Jill Urquhart
Mart Ustav
Thomas Vaccari
Jorge Valadas
Juan Valcarcel
Nicole Valenzuela
Vito Valiante
Celine Vallot
Cheryl van Buskirk

Daniel Van Damme
Yves van de Peer
Esther van de Vosse
Casper van der Kooi
Alexander van der Linden
Monique van der Voet
Marjan van der Woude
Mark Van Doren
Jessica van Setten
Bruno van Swinderen
Carter Van Waes
Josien van Wolfswinkel
Stepanka Vanacova
Ales Vancura
Daniel VanDamme
Wim Vandenberghe
Michiel Vandenbussche
Miri VanHoven
Thiago Venancio
Sandeep Venkataram
Kristen Verhey
Esther Verheyen
Evelyn Verhulst
Robert Verity
Paul Verslues
Patrik Verstreken
Kevin Verstrepen
Alfred Vertegaal
Michael Verzi
David Vetrie
Beatriz Vicoso
Andres Vidal Gadea
Sebastien Vigneau
Yves Vigouroux
Jean-Paul Vincent
Mark Viney
Rosella Visintin
Renaud Vitalis
Franco Vizeacoumar
Eszter Vladar
Bruce Vogel
Benjamin Voight
David Volz
Jan Willem Voncken
Ekaterina Voronina
Sophie Vriz
Paresh Vyas
Scott Waddell
Claire Wade
Michael Wade
William Wadsworth
Daniel Wagner
Doris Wagner
Samuel Wagner
Louise Wain
Yuichi Wakabayashi
Takayoshi Wakagi
John Wakeley
Virginia Walbot
Amy Walker
Russell Wallis
Jörn Walter
Klaudia Walter
Jian-Min Wan
Chaolong Wang
David Wang
Isabel Wang
Jia-Wei Wang
Jing Wang
John Wang
Maggie Haitian Wang
Meng Wang
P. Jeremy Wang
Ping Wang
Tao Wang
Wei Wang
Yong Wang
Yuh-Hwa Wang
Zhenghe Wang
Michael Wangler
Rui Wang-Sattler
Sjoerd Wanrooij
Andrew Ward
Alan Warren
Karen Wassarman
Yoshinori Watanabe
Jennifer Watts
Joshua Waxman

Vikki Weake
Daniel Weeks
Donate Weghorn
Pan Wei
Zhi Wei
Detlef Weigel
William Weis
Noah Weisleder
Robert Weiss
Bernd Weisshaar
Matthew Weitzman
Deneen Wellik
Raymund Wellinger
James Wells
Xiaoquan Wen
Zilong Wen
Hans-Guido Wendel
Juergen Wendland
Kerstin Wendt
Stephan Wenkel
Mathias Wernet
Benedikt Westermann
Pål Westermark
Anja Marie Westram
Marijke Wevers
Judy Wexler
Heather Wheeler
Stephen Whisson
Charles White
Frank White
Michael White
Alexander Whitworth
Claude Wicker-Thomas
Vihandha Wickramasinghe
Philipp Wiemann
Rudolf Wiesner
Annegret Wilde
Karl Willert
Amy Williams
David Williams
Valerie Williamson
Ian Willis
Richard Wilson
Samuel Wilson
Klaus Wimmers
Thomas Winkler
Fred Winston
Elizabeth Winzeler
Christopher Witt
Andreas Wodarz
Kenneth Wolfe
Mariana Wolfner
Jason Wong
Richard Wood
Will Wood
Michael Woods
Henry Wortis
Johannes Wostemeyer
Nicholas Wright
Stephen Wright
Baolin Wu
Gang Wu
Jianqiu Wu
Xiaohua Wu
Xifeng Wu
Zhe Wu
Wolfgang Wurst
Sarah Wyatt
Cheng-Bin Xiang
Xinshu Xiao
Chao Xing
Zhi-Qi Xiong
Hao Xu
Huaxi Xu
Jian Xu
Jianfeng Xu
Lifeng Xu
X. Z. Shawn Xu
Chaoyang Xue
Ding Xue
Katherine Xue
Koji Yahara
Gen Yamada
Masamitsu Yamaguchi
Ayumu Yamamoto
Keith Yamamoto
Takashi Yamashiro
Yukiko Yamashita

Kazuki Yamazawa
Hua Yan
Jianbing Yan
Jie Yan
Riqiang Yan
Wei Yan
Can Yang
Chengwei Yang
Jing Yang
Jingjing Yang
Jun Yang
Lixing Yang
Wei Cai Yang
Wei Yang
Xiangdong William Yang
Yufeng Yang
Marcelo Yanovsky
Judith Yanowitz
Humphrey Yao
Song Yao
Dag Yasui
Yasuo Yasui
Trevor Yeats
Lamis Yehia
Pamela Yelick
Foong Yeong
Ruohe Yin
Yanhai Yin
Keiichiro Yogo
Gyeong Mee Yoon
Keiko Yoshioka
Young-Jai You
Kevin Young
Marian Young
Matthew Young
Hongtao Yu
Jianming Yu
Kai Yu
Fang Yuan
Jiao Yuan
Ling Yuan
Katherine Yutzey
Hani Zaher
Ronen Zaidel-Bar
Arslan Zaidi
Noah Zaitlen
Jozsef Zakany
Mostafa Zamanian
Phillip Zamore
David Zappulla
Kathi Zarnack
Frank Zaucke
Ricardo Zayas
Phillip Zegerman
Martin Zeidler
Kai Zeng
Martin Zenker
Dabing Zhang
Fangyuan Zhang
Gao Zhang
Ge Zhang
Jianqi Zhang
Liangran Zhang
Rui Zhang
Xian Sheng Zhang
Xin Zhang
Xiuren Zhang
Xuemei Zhang
Yan Zhang
Yong Zhang
Yuelin Zhang
Zhenguo Zhang
Jing Hua Zhao
Li Zhao
Xiaolan Zhao
Yunde Zhao
Deyou Zheng
Jie Zheng
Yixian Zheng
Degui Zhi
Xiaomin Zhong
Bin Zhou
Huilin Zhou
Jian-Min Zhou
Qi Zhou
Xiang Zhou
Zhemin Zhou
Zhixiong Zhou

Jian-Kang Zhu
Nan Zhu
Anna Zinovyeva
Robert Zinzen
Piotr Ziolkowski
Sebastian Zollner
Leonard Zon
Jessica Zucman-Rossi
Jian Zuo
